# Air Pollution Status in 10 Mega-Cities in China during the Initial Phase of the COVID-19 Outbreak

**DOI:** 10.3390/ijerph18063172

**Published:** 2021-03-19

**Authors:** Crystal Jane Ethan, Kingsley Katleho Mokoena, Yan Yu

**Affiliations:** Health Science Center, School of Public Health, Xi’an Jiaotong University, Xi’an 710061, China; crystal2016@stu.xjtu.edu.cn (C.J.E.); kk.mokoen@xjtu.edu.cn (K.K.M.)

**Keywords:** air pollution, COVID-19, mega-cities, particulate matter, air quality

## Abstract

Over the past decades, urbanization and industrialization have led to a change in air quality, bringing researchers to a full realization of the damaging effects of human activities on the environment. This study focused on describing air quality during the initial phase of the Novel Coronavirus disease (COVID-19) pandemic (since there were fewer anthropogenic activities) in 10 Chinese mega-cities. Using the independent *t*-test, the means of air quality index (AQI) scores and individual air pollutants concentration during the outbreak were compared with the means before the outbreak. Cohen’s d was estimated to quantify how much difference was observed. Based on the AQI score, the air quality in these 10 cities ranged from excellent (Shenzhen) to light pollution (Xi’an) with 44.8 μg m^−3^ and 119.7 μg m^−3^, respectively. In comparison to the 2019 air quality, Guangzhou and Wuhan noted major differences in air quality during the outbreak. Indicators of traffic pollution, particularly NO_2_, were significantly lower during the outbreak in all cities. Particulate matter pollution varied, with some cities observing lower concentrations and other higher concentrations during the outbreak. There was a significant decrease in air pollution levels during the outbreak. More researchers should observe changes in air quality during peculiar or major events. Implementation of stringent regulation on vehicle use should be considered in mega-cities. Relevant findings should be employed in emphasizing the detrimental effects of anthropogenic activities and support the need for stringent emission control regulations.

## 1. Introduction

In the last three decades, the level of pollution in China has increased exponentially due to extensive urbanization and mass industrialization. However, this rapid economic rise feat also came with major environmental challenges, including degradation and pollution (air, soil, and water). Regarding air pollution, six major pollutants are considered as criteria air pollutants; these include fine particulate matter (PM_2.5_), inhalable particulate matter (PM_10_), ozone (O_3_), sulfur dioxide (SO_2_), nitrogen dioxide (NO_2_), and carbon monoxide (CO). Particulate matter (PM_2.5_ and PM_10_) refers to microscopic solid or liquid matter that is suspended in the atmosphere. Both are typically derived from either natural sources (volcanoes, dust storms, sea sprays, etc.) or anthropogenic sources (fossil fuel, coal combustion) and differ only in their sizes. Ozone is an inorganic gaseous molecule occurring from natural sources (lightning) or the reaction between solar radiation and nitrogen oxides (NOx) or volatile organic compounds. Sulfur dioxide is a toxic gas derived from natural sources such as volcanoes, or anthropogenic sources (the combustion of sulfur-containing fossil fuels and the oxidation of organosulfur compounds). Nitrogen dioxide is a gaseous compound emitted from the combustion of fossil fuels (vehicles engines, gas cookers, and heaters). Carbon monoxide is an odorless, colorless gas that is emitted by combustion engines, such as cars, stoves, furnaces, etc. [1]. These air pollutants pose a health hazard for individuals of all gender and ages, particularly those with compromised immune systems. China is home to some of the world’s biggest and most populous cities (i.e., mega-cities), which often suffer severe air pollution due to major industrial activities, continuous construction and infrastructural development, and high traffic volume [2].

Beyond a reasonable doubt, air pollution in China presents a serious health threat, as shown in several ecological studies. Studies carried out in major Chinese cities have associated air pollution exposure with illnesses ranging from common influenza to chronic diseases, including mortality. To note a few, Shang et al. revealed overall daily mortality was associated with exposure to ambient air pollution in several Chinese cities; Cao et al. linked fine particulate matter and cardiopulmonary mortality in heavily polluted Chinese cities; Mokoena et al. linked respiratory mortality and air pollution in Xi’an city; and Xu et al. associated ischemic heart disease with particulate matter pollution in Beijing [2,3,4,5]. The risk of air pollution exposure for citizens in urban areas remains high in comparison to their rural counterparts. Moreover, if the air quality changes observed during the 2008 Beijing Olympics and the annual Chinese New Year Celebrations are used as evidence, it shows that major events tend to affect air pollution levels [6,7,8]. The hike or reduction in air pollution levels can be attributed to change in traffic volume and industrial activities [6]. This directly proves that anthropogenic activities are major contributors to air pollution levels in urban centers [9,10].

In December 2019, an outbreak of COVID-19, caused by severe acute respiratory syndrome coronavirus-2 (SARS-CoV-2), was first reported in the Chinese city of Wuhan in Hubei Province and quickly spread throughout China, leading to a complete lockdown of Wuhan (the epicenter) and curfews in other Chinese cities for several weeks [11]. The severity of the epidemic led to travel restrictions, closure of workplaces including industries, suspension or cancellation of schools, major events, and outdoor activities. The observance of the imposed shutdown and restricted movement in most cities left a positive impact on the environment. Several satellite images showed an obvious reduction in air pollution levels in China [12,13]. Therefore, the current study aims to describe air quality and criteria air pollutants concentration level during the COVID-19 lockdown period (January to March 2020) in 10 Chinese mega-cities. The study hypothesizes that there is no difference between air quality or air pollution concentration level before and during the COVID-19 lockdown periods.

## 2. Materials and Methods

### 2.1. Data

**Figure 1:**
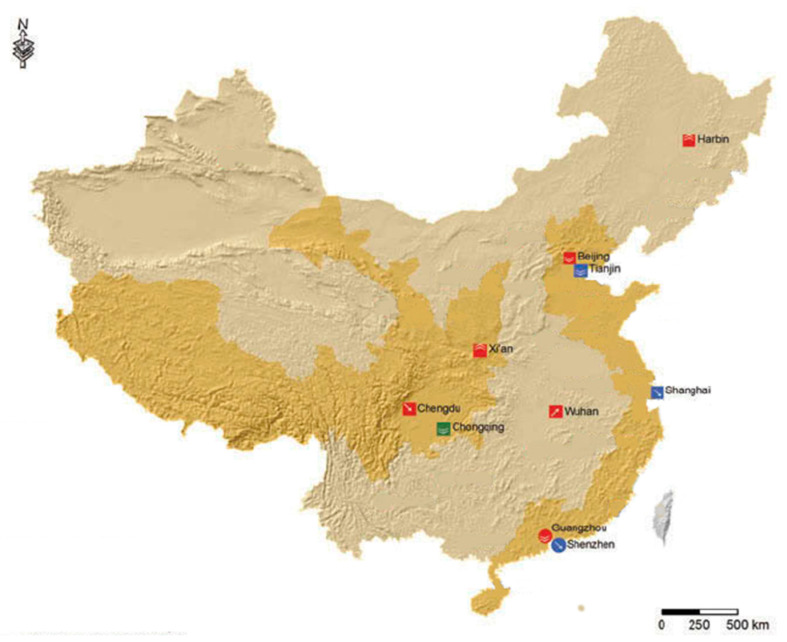
Map of China displaying the 10 mega-cities observed in the study. The map was adopted from Zhou et al. “Effects of spatial form on urban commute for major cities in China,” and modified for use in this publication [14].

The study area included 10 mega-cities in China, namely, Beijing, Shanghai, Xi’an, Chongqing, Wuhan, Guangzhou, Harbin, Chengdu, Tianjin, and Shenzhen (Figure 1) [14]. These cities are densely populated, ranging from 10 million (Harbin) to 30 million (Chongqing) people. All cities observe the same four seasons with slight differences, such as extreme winter in Harbin and warmer winter in Guangzhou. Their main sources of air pollutant emission are also similar, namely, coal combustion for heating, combustion of fossil fuels from vehicles, and industrial fumes. All cities also observed similar restrictions during the outbreak, namely, closure of offices, manufacturing industries, educational institutions, commercial centers, leisure places, etc.

Air quality index score and air pollutants’ concentration levels were collected for 10 major cities in China. The data covered the initial period of the outbreak and a year before the outbreak. Daily records for AQI (μg m^−3^), PM_2.5_ (μg m^−3^), PM_10_ (μg m^−3^), O_3_ (μg m^−3^), SO_2_ (μg m^−3^), NO_2_ (μg m^−3^), and CO (mg m^−3^) were collected from AQI China—a national online database. The main data covered from 1 January 2020, to 31 March 2020 (initial COVID-19 outbreak period). In addition, similar data were collected for the previous year, from 1 January 2019 to 31 December 2019. All data were generated from environmental monitoring stations within each city. No dates were missing. Note that all air pollutants were measured in microgram per cubic meter (μg m^−3^) except CO, which was measured in milligram per cubic meter (mg m^−3^).

### 2.2. Statistical Analysis

The main statistical test employed was the independent samples *t*-test.

#### 2.2.1. First Phase: Data Categorization

To categorize the data into meaningful groups, the time of the year during which the outbreak occurred and the seasons (winter, autumn, spring, and summer) observed in China were taken into consideration. This is based on the fact that seasonality has been noted to influence air pollution levels [2]. The entire data retrieved from January 2019 to March 2020 was divided into five categories as follows:

Category A—1 month to 3 months before the outbreak (October 2019 to December 2019);
Category B—3 months to 6 months before the outbreak (July 2019 to September 2019);
Category C—6 months to 9 months before the outbreak (April 2019 to June 2019);
Category D—9 months to 12 months before the outbreak (January 2019 to March 2019);
Category K (constant)—during the outbreak (January 2020 to March 2020).

#### 2.2.2. Second Phase: Descriptive Analysis

A descriptive analysis gives a summary of the data. It is divided into two main categories, namely, (i) measure of central tendency (mean and median) and (ii) measure of variability (standard deviation, percentile, and ranges) [15]. The descriptive analysis was carried for all data (AQI scores and individual air pollutants).

#### 2.2.3. Third Phase: Independent *t*-Test Analysis

The independent *t*-test, also referred to as the two-sample *t*-test, is an inferential statistical analysis that determines if there is a statistically significant difference between the means of two unrelated groups. To carry out a *t*-test, the dependent variable should assume a normal distribution [16]. In this regard, the data used in the study met the assumption of normality; this was possible due to the size of the data (for each category *n* = 90, 91, or 92; holistically *n* = 456). According to Piovesana and Senior, a sample size of 50 is sufficient to obtain normality, while a sample size greater than 85 is sufficient to obtain a stable means and standard deviation regardless of the level of skewness [17]. Therefore, the size of data in this study was sufficient to accord normality. Moreover, the independent variables must be unrelated. This criterion was met on the basis that air pollution concentration on lockdown days and non-lockdown days cannot be ideally the same (since the dates).

The following comparisons were employed in the analysis: category A vs. category K; category B vs. category K; category C vs. category K and category D vs. category K. The mean difference was estimated as follows: before the outbreak (category A–D) and during the outbreak (category K). The *t*-score and *p*-value were also derived from the test. Statistical analysis was carried out using IBM SPSS 24.0 (IBM, Armonk, NY, USA).

#### 2.2.4. Fourth Phase: Estimation of Effect Size

For further assurance that the differences observed from the *t*-test are reliable, the effect size (i.e., the standardized difference between two means) was estimated using Cohen’s d [18].

Cohen’s d is given by Equation (1):

(1) $$d=\frac{M_{1}-M_{2}}{S_{pooled}},$$

where *M_1_* and *M_2_* are the means for groups 1 and 2 respectively and *S_pooled_* is the pooled standard deviation for the two groups. Cohen’s d was calculated using the Rstats effect size calculator [19]. In addition, 1 Cohen’s d = 1S (standard deviation); 1S = 1 z score; and 1 z-score is equivalent to a defined value on the z-score table. The Rstats normal distribution table was used to determine the defined value of the z-scores. The final value shows, in reality, by how many points one group’s mean is lesser or higher than another (mean ± z) [20]. Cohen’s d can be interpreted using its rule of thumb (convention). Cohen and Sawilowsky’s convention for effect size (d ≤ 0.01—very small; d = 0.01–0.2—small; d = 0.2–0.5—medium; d = 0.5–0.8—large; d = 0.8–1.2—very large; d = 1.2–2—huge) equate effect sizes to the amount of actual difference between two groups [18,21].

## 3. Results

### 3.1. Descriptive Statistics

Table 1 shows some characteristics of AQI during the outbreak/lockdown period. The average AQI scores for the 10 cities ranged from 44.8 μg m^−3^ (Shenzhen) to 119.7 μg m^−3^ (Xi’an). Although Xi’an recorded the highest average AQI score over the lockdown period, Harbin, however, recorded the highest daily AQI score of 327 μg m^−3^ and the most dispersed measure of air pollution concentration (SD = 78.9; range = 289). Shenzhen on the other hand maintained the least average (44.8 μg m^−3^) and daily (19 μg m^−3^) AQI scores and the least dispersed measure of air pollution concentration (SD = 10.8; range = 56) over the outbreak period. Table S1 (Supplementary Materials) shows descriptive statistics for individual air pollutants. Shenzhen and Harbin recorded the least (22.5 μg m^−3^) and highest (85.9 μg m^−3^) average PM_2.5_ concentrations during the lockdown, respectively. Shenzhen and Xi’an recorded the least (37.7 μg m^−3^) and highest (117.5 μg m^−3^) average PM_-10_ concentration, respectively. Beijing and Harbin recorded the least (4.8 μg m^−3^) and highest (28.6 μg m^−3^) average SO_2_ concentration, respectively. Shenzhen had the least average CO concentration of 0.6 mg m^−3^, while Xi’an, Wuhan, and Tianjin had the highest average CO concentration of 1.0 mg m^−3^ each. Shenzhen and Tianjin recorded the least (21.9 μg m^−3^) and highest (43.9 μg m^−3^) average NO_2_ concentration, respectively. Chongqing and Shanghai recorded the least (51.8 μg m^−3^) and highest (84.1 μg m^−3^) average O_3_ concentration, respectively.

Table 2 shows the descriptive statistics (central tendency) of AQI scores before the outbreak; similar results for individual air pollutants are available in the Supplementary Material (Table S2). AQI scores and individual air pollutant concentration levels in all cities varied prior to the outbreak. Cities such as Xi’an, Harbin, and Tianjin, which recorded high AQI scores during the outbreak also had similar scores before the outbreak.

### 3.2. Graphical Presentation of AQI Scores and Key Air Pollutants Concentration Level

**Figure 2:**
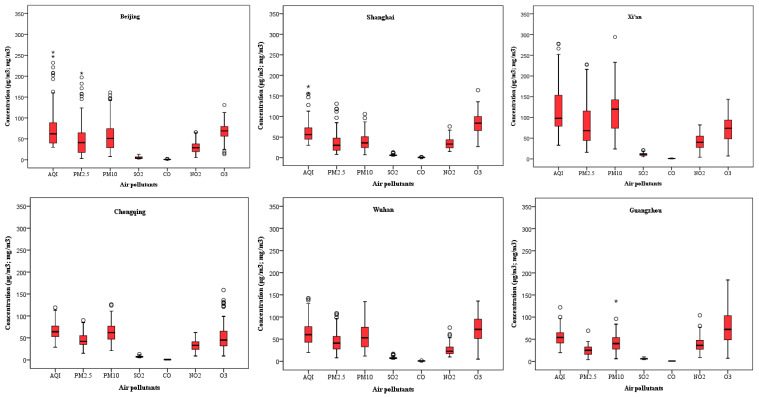

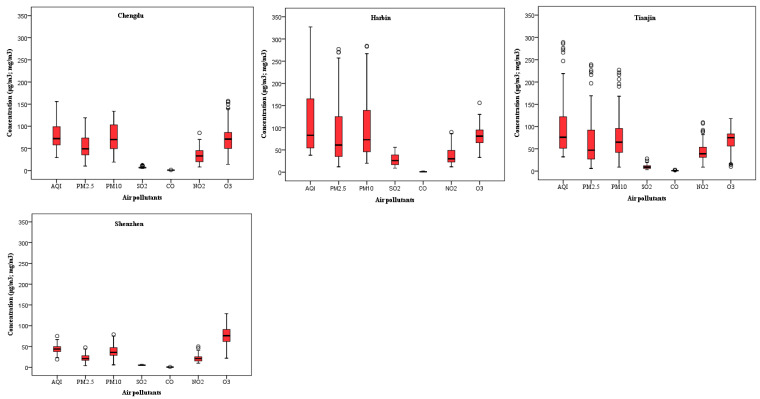
A visual summary of AQI scores and air pollutants concentration in 10 major Chinese cities during the initial phase of the COVID-19 outbreak (lockdown period), showing the minimum, median, maximum, lower quartile, and upper quartile value of each parameter.

Figure 2 graphically displays some characteristic features of AQI scores and individual air pollutants concentration in the 10 cities observed. The figure also reveals Beijing, Shanghai, and Tianjin had more outliers and extreme values than other cities, indicating there were several days of extreme pollution within these cities. All cities except Chengdu had outliers for particulate matter pollution (PM_2.5_ and PM_10_), while Beijing and Guangzhou had extreme values for inhalable particulate matter only. There were more outliers for particulate pollution than gaseous pollution (O_3_, SO_2_, NO_2_, and CO) in the 10 cities observed. In other words, there were more days of extreme particulate pollution than gaseous pollution.

**Figure 3:**
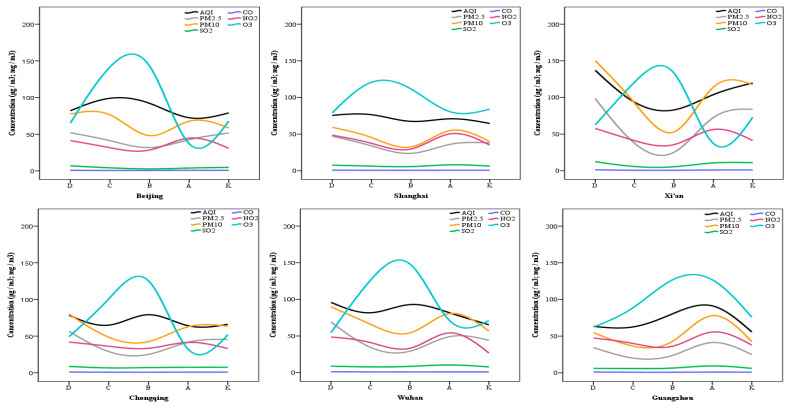

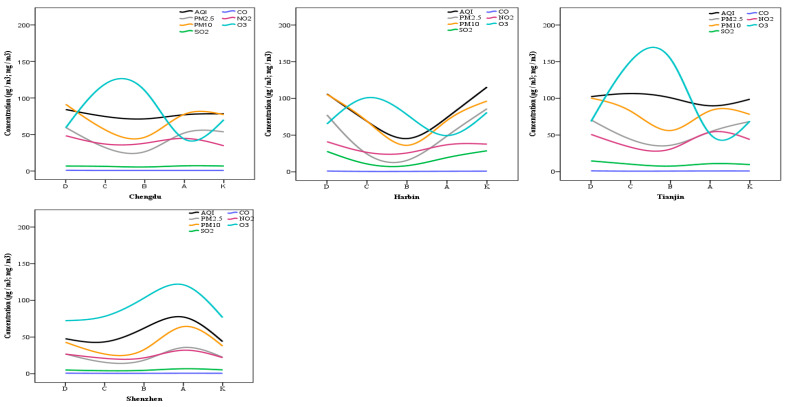
Graphical display of air pollution level in 10 major Chinese cities several months before and during the initial phase of the COVID-19 outbreak (lockdown period), highlighting the difference in air quality at different periods. A—1 month to 3 months before the outbreak (October 2019 to December 2019); B—3 months to 6 months before the outbreak (July 2019 to September 2019); C—6 months to 9 months before the outbreak (April 2019 to June 2019); D—9 months to 12 months before the outbreak (January 2019 to March 2019); K (constant)—during the outbreak (January 2020 to March 2020).

Figure 3 displays the quality of air in the 10 cities before and during the COVID-19 outbreak based on average AQI scores and air pollutants concentration level. The patterns for all air pollutants were similar in the 10 cities. PM_2.5_, PM_10_, NO_2_, and SO_2_ observed high concentration levels from 9 months to 12 months before the outbreak, followed by a decrease from 9 months to 3 months before the outbreak, a slight increase from 1 month to 3 months before the outbreak, and a final decrease during the outbreak. A closer look at the matching months (January–March 2019 and 2020) provides a definite revelation that the lockdown to a great extent curtailed the emission of these air pollutants; hence, there was a lower concentration of air pollution. Ozone on the other hand had an opposite pattern; it noted low concentration from 9 months to 12 months before the outbreak, followed by a major increase from 9 months to 3 months before the outbreak, then a decrease from 1 month to 3 months before the outbreak, and a slight increase during the outbreak. This was noted for all cities except Shenzhen and Guangzhou; O_3_ concentration in both cities did not increase during the lockdown—it dropped considerably in comparison to three months before the lockdown. Focusing only on matching months (January–March 2019 and 2020), the graph shows that ozone concentration in all cities had a minimal difference between both time frames. Carbon monoxide had an almost linear pattern prior to and during the outbreak.

### 3.3. Description of Air Quality

Based on the average AQI analyzed, air quality in each city before and during the outbreak was labeled according to the standard AQI range (China’s and WHO air quality guidelines), that is, excellent: 0–50 μg m^−3^, good: 51–100 μg m^−3^, light pollution: 101–150 μg m^−3^, moderate pollution: 150–200 μg m^−3^, heavy pollution: 201–300 μg m^−3^, and severe pollution: >300 μg m^−3^ [10]. Table 3 shows the corresponding description for the average AQI scores estimated for each city. The air status during the outbreak ranged from excellent (Shenzhen) to light pollution (Xi’an and Harbin). In comparison to the few months before the outbreak and the same interval of the previous year (January–March 2019), little difference is noted. According to the description presented below, air quality in many cities remained the same during the outbreak as they were before the outbreak; this is only because the description of AQI relies on a range of numbers and not a specific number. However, in-depth statistical analysis of individual air pollutants shows there are differences between the air quality before and during the outbreak.

### 3.4. Independent t-Test Result

Table 4 shows the estimated mean difference and its 95% CI, t score, and effect size for comparison of air pollution averages during the lockdown and before the lockdown.

A statistically significant difference in air pollution concentration was observed for comparison between the shutdown period and prior to the shutdown; however, this was not noted in all 10 cities. Therefore, we reject the null hypothesis. The differences between both periods were positive (indicating AQI was lower during the outbreak) in some cities, while others were negative (indicating AQI was higher during the outbreak). The air quality in Beijing, Shanghai, and Shenzhen during the lockdown was significantly different when compared to their air quality in only a few months in 2019. Harbin and Xi’an noted a different air quality during the outbreak in comparison to most months in 2019, while Guangzhou and Wuhan had significantly different air quality during the lockdown in comparison to the air quality throughout 2019. Tianjin observed no statistically significant difference between its air quality during the outbreak and in 2019.

## 4. Discussion

The descriptive summary highlights certain features of air pollution concentration in each city. Firstly, the most polluted city observed in this study, during the shutdown period was Xi’an, followed by Harbin, Tianjin, Beijing, and Chengdu. (Table 1). Nevertheless, it does not imply that Xi’an, Harbin, or Tianjin experienced more air pollution emissions during the lockdown than other cities. The reason is that these three cities are known for usually experiencing steeper air pollution than others. Hence, even with a lockdown, the reduction of air pollution concentration might not be very drastic to bring about better air quality than cities that usually have less air pollution. The rationale behind this is that air pollutants (especially particulate matter) last several days to weeks in the atmosphere [22]. Therefore, the typical air quality of a city played a role in determining the air quality observed during the lockdown. The descriptive analysis also revealed several cities recorded spikes in air pollution concentration levels. However, it made no difference in the ranking of air quality for each city. Since the mere description of the air pollutants concentration does not shed sufficient light on the quality of air during the COVID−19 outbreak, a comparison of air quality during different periods prior to the outbreak was logical to derive a broader and better understanding [22,23].

From the independent *t*-test analyses, a positive mean difference indicates the AQI (μg m^−3^) score during the outbreak was lower than the score before the outbreak. Positive mean differences were noted in nine cities for comparison of air quality during the lockdown and category D, (January–March 2019); in five cities for category C (April–June 2019); in seven cities for category B (July–September 2019) and in four cities for comparison with category A (October–December 2019) (Table 4). However, these are only statistically important if the estimates are statistically significant.

In the interpretation of results, it is crucial to be cautious and take into consideration the typical concentration levels of air pollutants at certain periods of the year. For instance, ozone is typically low during cold periods and high during warm periods (Figure 3). Hence, an expected difference would be noted between concentration levels during the outbreak and prior to the outbreak (April–September, since these months typically have high ozone concentration). Therefore, we might consider an actual decrease in ozone concentration during the outbreak (January–March 2020) when compared with the concentration level during the same period of the previous year (January–March 2019) simply because both periods observe the same meteorological characteristics (i.e., high wind speed, low relative humidity, low temperature, high atmospheric pressure, etc.); hence, any difference observed is void of time/season bias. As shown above (Figure 3), ozone concentration from the matching months had very minimal difference. A similar rationale goes for particulate matter, which is notably low in warm months and high in cold months. Thus, focusing on the comparison of matching months gives a simple yet accurate understanding of any difference noted. Nonetheless, if air pollutants concentration that should have been typically low during the outbreak surpassed the concentration level of previous months (especially for months that typically have a high concentration level), this indicates air pollution during the outbreak was remarkably high. In this regard, no air pollutant was noted.

Based on the average AQI score, Beijing’s overall air quality during the initial phase of the outbreak (lockdown) did not differ significantly from air quality of the same months in 2019 (January–March). However, analysis of individual air pollutants reveals that PM_10_, SO_2_, and NO_2_ concentrations during the lockdown were significantly different (lower) than concentrations for the same months in 2019. For comparisons with other months in 2019, overall air quality in Beijing during the lockdown differed significantly from air quality between April–June of 2019. The effect size (0.411) and the corresponding value (0.1591) indicate the difference was small. Moreover, NO_2_ pollution dropped drastically during the outbreak compared to its concentration between October–December 2019; its effect size was large (0.811) (Table 4 and Table S3–Supplementary Materials). This was similar to the observation of NO_2_ pollution during the Beijing 2008, Olympics [8]. In Shanghai, the average AQI during the outbreak was significantly lower than the average AQI recorded between January 2019 to March 2019 and April 2019 to June 2019, with effect sizes of 0.394 and 0.396, respectively. Particulate matter pollution was significantly lower during the lockdown than in the same months in 2019, although PM_10_ had a larger effect size (0.792) than PM_2.5_ (0.348). CO and NO_2_ also noted a small and large difference, respectively, during the outbreak in comparison to the matching months. Overall, air pollution in Xi’an during the lockdown showed no statistical difference from pollution between January 2019 to March 2019. The same outcome was noted for PM_2.5_ concentration, insinuating that the shutdown period did not affect Xi’an’s fine particulate matter pollution and its overall air pollution. To buttress this point further, the average AQI score and PM_2.5_ concentration in Xi’an during the outbreak were significantly higher than the concentration observed from April 2019 to September 2019. However, observation of other air pollutants revealed that there was a large difference between NO_2_ pollution during the outbreak and before the outbreak (precisely between January–March 2019 and October–December 2019), indicating traffic-related pollution was reduced. Using Magnusson’s interactive visualization of effect size reveals that 81.9% and 83.4% of daily NO_2_ concentration recorded during the outbreak was lower than daily NO_2_ concentration for both periods (Table 4 and Table S3—Supplementary Materials) [24]. In Chongqing, overall air quality during the lockdown differed significantly from air quality for matching months in 2019. Individual air pollutants such as fine and inhalable particulate matter were also lower during the outbreak in comparison to the same period. For other months in 2019, particulate pollution was significantly higher during the outbreak. However, that is expected considering particulate matter concentration increases during cold seasons and reduces during warm seasons. As with particulate pollution, gaseous pollution was also lower during the outbreak than between January–March 2019; the significant effect sizes for SO_2_, CO, and NO_2_ were 0.559, 0.512, and 0.770, respectively.

Wuhan, which was the epicenter of the COVID-19 outbreak in China, experienced a thorough and longer shutdown period than other cities [25,26]. Consequently, the air quality improved because individual air pollutants concentration levels decreased. The average AQI in Wuhan during the shutdown period was lower than the average AQI observed from January 2019 to December 2019. The effect sizes of the difference ranged from medium (0.565) to large (0.884) (Table 4). As indicated by the effect sizes, PM_2.5_ (0.862) and PM_10_ (1.034) noted large differences during the shutdown period when compared with particulate pollution from January 2019 to March 2019. Additionally, indicators of traffic-related pollution showed a decrease in concentration level during the outbreak. NO_2-_, in particular, showed a very large significant decrease in concentration levels when compared to levels noted for January 2019 to June 2019 and October 2019 to December 2019. Similar to Wuhan, the average AQI in Guangzhou during the outbreak was significantly lower than before the outbreak (for all of 2019). CO and NO_2_ concentrations were significantly different when compared to concentrations for January 2019 to March 2019. Particulate matter pollution observed a very large decrease during the outbreak in comparison to its pollution between October–December 2019 only. Opposite to Guangzhou, the average AQI and concentration of most air pollutants in Harbin were significantly higher during the outbreak than before the outbreak (for all of 2019). Only ozone concentration was significantly lower during the lockdown when compared to the concentrations between April 2019 to June 2019. Particulate matter pollution during the lockdown had no statistically significant difference with its pollution between January 2019 to March 2019. Its concentration level between July 2019 to September 2019 was also lower than during the outbreak, with an effect size of −1.466 and −1.253 for PM_2.5_ and PM_10_, respectively. Given that Harbin experiences extreme winter, PM_2.5_ levels are often high in winter since the combustion of coal used for heating purposes is a major source for PM_2.5_ emission. This has been noted in some previous studies [27]. In light of this fact, we can deduce the shutdown had no significant effect (decrease) on particulate pollution since no statistical difference was observed when compared to January–March 2019. Tianjin’s average AQI during the outbreak was not significantly different from before the outbreak. However, PM_10_, SO_2_, and NO_2_ noted lesser concentration levels during the outbreak. The decrease was significantly different in comparison to concentration levels between January 2019 to March 2019. For comparison with concentration levels between April 2019 to September 2019, only ozone showed a significant difference. In Shenzhen, air quality during the outbreak was only significantly different for comparison to categories B and C. The indicator for the air quality during the outbreak was more or less the same as all January to March periods. Nonetheless, during the outbreak, individual air pollutants—PM_10_, CO, and NO_2_—noted small (0.293), medium (0.479), and large differences (0.833), respectively, when compared with their counterparts for January–March 2019. Other air pollutants were higher during the outbreak than before it.

Overall, the following changes in air quality were observed. The concentration levels for indicators of traffic-related air pollution (NO_2_ and CO) were remarkably reduced in most cities during the outbreak. The major reason for this amount of decrease is that the restriction on the outdoor movement led to fewer cars being on the road, directly resulting in lesser emission of these pollutants. In a preliminary analysis, National Aeronautics and Space Administration (NASA) researchers compared NO_2_ concentration in early 2020 with the average concentration detected at the time of year from 2005–2019. They noted NO_2_ concentrations in 2020 in eastern and central China were significantly lower (from 10% to 30% lower) than what is normally observed for that period [28]. As of 2018, China’s car parc stood at 240 million (car parc refers to the number of vehicles in use in a region) [29]. The lockdown ultimately caused a large percentage of these cars to be off the road. The decrease in traffic-related air pollution has several health advantages, many of which are related to respiratory wellness (given that pollutants such as NO_2_ are absorbed entirely along the entire respiratory tract and are deposited peripherally in the lungs). NO_2_ is known to induce inflammation of the airway after at least one exposure; precisely, bronchial responsiveness occurs at concentrations of ≥1800 μg m^−3^ in healthy persons and ∼200–500 μg m^−3^ in asthmatic patients or those with chronic obstructive pulmonary disease (COPD). The pollutant also amplifies the asthmatic response to allergen exposure. In highly trafficked areas, 15 minutes at 500 μg m^−3^ exposure can intensify allergic inflammatory reactions in the airways without causing symptoms or pulmonary dysfunction [30]. Therefore, a drastic reduction in traffic pollution is of great health benefits to both healthy and compromised individuals.

Since industrial activities (such as oil shale mining, power generation, steel mining, etc.) and the use of heavy machinery had come to a standstill in compliance with the shutdown. A major indicator of industrial pollution (SO_2_) also noticed a moderate reduction in concentration level during the outbreak. Satellite imagery shows cities such as Shanghai and Wuhan noted a 31.3% and 3.9% reduction in SO_2_, respectively [31,32]. Another study covering 366 urban areas in China revealed SO_2_ concentration had dropped by 12% between January 2020 to April 2020, in comparison to concentration at the same time in 2019 [33]. Although moderate, the decrease in SO_2_ is advantageous to health and the economy. According to Zen et al., industrial air pollution causes an increase in medical expenses. Therefore, any decrease noted from its major constituent will directly or indirectly lower medical budgets [34]. Regarding health, residents of industrial areas more frequently reported wheezing, chest tightness, shortness of breath, hypertension, heart diseases, etc. than non-industrial residents [35].

In contrast, particulate pollution, especially PM_2.5_, did not note much decrease in concentration in most cities. The study by Silver et al. noted that PM_2.5_ and PM_10_ concentrations in China were lower by 10.5% and 21.4%, respectively, during the lockdown [36]. This is understandable, given that the combustion of coals for heating purposes and the use of indoor burners did not stop during the shutdown. Additionally, the secondary particulate matter, which is generated from reactions of sulfur dioxide and nitrogen dioxide, was still emitted, even though their sources were reduced. Hence, a fairly high concentration of particulates in the atmosphere was maintained during the entire period of the shutdown. In as much as every decrease in particulate matter pollution is seen as positive, a difference in health can only be achieved if concentration levels are within the recommended health limits. Some sources have estimated 3–5 μg m^−3^ as the lowest concentration level at which adverse effects due to PM_2.5_ start to manifest [23]. At or above these levels, particulates can penetrate the bloodstream, lungs, tissues, and other organs, causing a wide range of adverse health effects. According to Cohen et al., particulate matter causes about 3% of mortality from cardiopulmonary disease, about 5% of mortality from cancer of the trachea, bronchus, and lung, and about 1% of mortality from acute respiratory infections in children under five years old worldwide. [37]. Unlike other air pollutants, ozone noted a consistent increase (though small) in most cities during the lockdown. Given that it is a secondary pollutant (from solar radiation and NO_x_), it would be expected that a decrease in NO_2_ would also facilitate a decrease in ozone. Unfortunately, this was not the case. A study on China’s air quality attributed O_3_ increase to nitrogen oxide titration effect (in the reduction of NO_x_ (NO_2_ + NO), O_3_ is removed in the presence of high concentrations of NO [36]. This outcome does not favor the public, especially the elderly, given that the effect of ozone has been established to be more prominent in this category of people. Its adverse effect on health ranges from mild respiratory illnesses to circulatory diseases to chronic disorders [3].

The study has strengths and limitations. This is a novel study describing the impact a major event or occurrence can have on the air. In plain terms, the study highlights the effect of daily activities (such as the use of vehicles) on air quality. The study creates a window of opportunities for different related studies (for example, understanding the health implications of the change in air quality). Aside from its strength, the study is limited in few aspects. Firstly, all cities in the study did not commence the shutdown on the same date and with the same intensity. Secondly, the analysis only describes the quality of air in these cities; the data were not correlated with health data to assess exposure risk.

## 5. Conclusions

In conclusion, there was a difference in air quality (an improvement) for some cities during the outbreak, indicating that the shutdown positively affected the air quality status in these cities. Exploration of individual air pollutants further revealed that the kind of event or circumstance will determine which air pollutants concentration increases or decreases. Additionally, people’s daily activities contribute immensely to the quality of air in our environment. Hence, there is a need for more observations of air quality during certain periods (for example, festive seasons) to determine strategies that will be useful in curtailing the emission of air pollutants.

## Figures and Tables

**Table 1:** Descriptive statistics for air quality index (AQI; μg m^−3^) in 10 mega-cities during the COVID-19 outbreak.

City	Mean(SD)	Median	Min	Max	IQR	Range	P25	P75	Skewness	Kurtosis
Beijing	79.1 (53.4)	62	30	257	49	227	40	89	1.80	2.67
Shanghai	64.5 (28.5)	56	30	173	28	143	45	73	1.82	3.53
Xi’an	119.7 (60.3)	98	33	278	77	245	78	155	0.91	0.13
Chongqing	66.1 (20.0)	64	29	119	24	90	53	77	0.49	−0.03
Wuhan	65.4 (28.3)	60	20	142	35	122	43	78	0.97	0.51
Guangzhou	55.6 (19.4)	54	20	122	24	102	41	65	0.63	0.53
Chengdu	78.3 (28.2)	72	29	156	42	127	58	100	0.60	−0.27
Harbin	115.5 (78.9)	83	38	327	113	289	52	165	1.12	0.23
Tianjin	98.8 (65.0)	76	32	289	71	257	51	122	1.44	1.41
Shenzhen	43.8 (10.8)	44	19	75	12	56	38	50	0.15	0.13

**Table 2:** Descriptive statistics for AQI (μg m^−3^) in 10 mega-cities before the COVID-19 outbreak.

City	1–3 Months b/o				3–6 Months b/o				6–9 Months b/o				9–12 Months b/o			
	Mean	Med	Min	Max	Mean	Med	Min	Max	Mean	Med	Min	Max	Mean	Med	Min	Max
Beijing	72.5 ^b^	67	23	233	92.1 ^a^	94.5	28	178	99.0 ^a^	94	36	195	82.0 ^a^	69.5	30	267
Shanghai	70.9 ^a^	65	20	148	67.4 ^a^	64	23	181	76.5 ^a^	68	36	202	75.5 ^a^	69	34	260
Xi’an	103.8	87	25	319	83.0 ^b^	84	32	150	93.5 ^b^	83	33	482	137.1 ^a^	110	54	346
Chongqing	64.2 ^b^	57	27	149	79.2 ^a^	72	32	203	64.9 ^b^	54	30	153	78.4 ^a^	74	28	175
Wuhan	81.7 ^a^	83	25	180	92.9 ^a^	91.5	35	171	81.7 ^a^	79	26	141	95.9 ^a^	89	28	214
Guangzhou	91.3 ^a^	87.5	38	164	80.4 ^a^	72	24	167	62.5 ^a^	57	36	168	63.3 ^a^	58.5	25	122
Chengdu	77.0 ^a^	67	32	183	71.4 ^b^	60	33	185	74.6 ^b^	67	30	171	84.2 ^a^	79	36	152
Harbin	73.9 ^b^	53	23	298	45.3 ^b^	42	18	103	68.8 ^b^	56	20	459	106.1 ^b^	86	35	414
Tianjin	89.9 ^a^	77	25	225	100.8 ^a^	98	28	191	106.6 ^a^	95	37	282	102.3 ^a^	80	33	298
Shenzhen	77.3 ^b^	73	41	180	62.1 ^a^	42	19	176	43.4 ^b^	38	20	110	47.7 ^a^	45	21	99

^a^ Average AQI scores higher than during the outbreak; ^b^ Average AQI scores lower than during the outbreak; b/o: before the outbreak.

**Table 3:** Air quality in 10 mega-cities during the COVID−19 outbreak, prior to the outbreak, and at a similar time in the previous year (based on average AQI score).

Period	During the Outbreak		1–3 Months b/o		9–12 Months b/o	
City	AQI (μg m^−3^) Score	Description	AQI (μg m^−3^) Score	Description	AQI (μg m^−3^) score	Description
Beijing	79.1	Good	72.5	Good	82.0	Good
Shanghai	64.5	Good	70.9	Good	75.5	Good
Xi’an	119.7	Light pollution	103.8	Light pollution	137.1	Light pollution
Chongqing	66.1	Good	64.2	Good	78.4	Good
Wuhan	65.4	Good	81.7	Good	95.9	Good
Guangzhou	55.6	Good	91.3	Good	63.3	Good
Chengdu	78.3	Good	77.0	Good	84.2	Good
Harbin	115.5	Light pollution	73.9	Good	106.1	Light pollution
Tianjin	98.8	Good	89.9	Good	102.3	Light pollution
Shenzhen	43.8	Excellent	77.3	Good	47.7	Excellent

b/o—before the outbreak.

**Table 4:** Independent *t*-test comparing the means of AQI (μg m^−3^) during and before the COVID-19 outbreak.

City	A vs. K			B vs. K			C vs. K			D vs. K		
	Mean Diff (95% CI)	*t*-Value	Effect Size	Mean Diff (95% CI)	*t*-Value	Effect Size	Mean Diff (95% CI)	*t*-Value	Effect Size	Mean Diff (95% CI)	*t*-Value	Effect Size
Beijing	−6.6 (−20.0, 6.8)	0.975	−0.144	13.0 (−0.9, 26.9)	1.847	0.273	19.9 (5.8, 34.1) ^c^	2.775	0.411 *	2.9 (−12.1, 17.9)	0.386	0.057
Shanghai	6.4 (−1.5, 14.3)	1.592	0.236	2.9 (−5.9, 11.7)	0.646	0.096	11.9 (3.1, 20.8) ^c^	2.657	0.394 *	11.1 (2.9, 19.2) ^c^	2.662	0.396 *
Xi’an	−15.8 (−33.4, 1.7)	−1.780	−0.236	−36.7 (−50.7, −22.7)	−5.163	−0.763 *	−26.2 (−43.7, −8.7)	−2.961	−0.439 *	17.5 (−1.8, 36.7)	1.791	0.266
Chongqing	−1.9 (−9.1, 5.3)	−0.526	−0.078	13.1 (3.9, 22.2) ^c^	2.835	0.419 *	−1.1 (−8.3, 6.0)	−0.313	−0.047	12.4 (4.8, 19.9) ^c^	3.223	0.479 *
Wuhan	16.4 (7.9, 24.8) ^c^	3.822	0.565 *	27.5 (18.3, 36.7) ^c^	5.893	0.871 *	16.3 (8.1, 24.6) ^c^	3.890	0.577 *	30.5 (20.4, 40.7) ^c^	5.944	0.884 *
Guangzhou	35.7 (28.2, 43.1) ^c^	9.414	1.392 *	24.8 (15.8, 33.9) ^c^	5.421	0.802 *	6.9 (1.0, 13.0) ^c^	2.266	0.336 *	7.7 (1.7, 13.7) ^c^	2.532	0.377 *
Chengdu	−1.3 (−10.8, 8.2)	−0.268	−0.040	−6.9 (−16.1, 2.3)	−1.486	−0.220	−3.7 (−12.2, 4.8)	−0.854	−0.127	5.9 (−2.1, 13.9)	1.464	0.218
Harbin	−41.6 (−61.7, −21.5)	−4.089	−0.605 *	−70.1 (−86.6, −53.5)	−8.324	−1.231 *	−46.7 (−66.2, −27.1)	−4.705	−0.697 *	−9.4 (−31.0, 12.1)	−0.860	−0.128
Tianjin	−8.9 (−25.1, 7.2)	−1.090	−0.161	2.0 (−14.0, 17.9)	0.243	0.036	7.7 (−8.8, 24.3)	0.923	0.137	3.5 (−15.6, 22.4)	0.368	0.055
Shenzhen	−15.8 (−33.4, 1.7)	−1.780	1.751	−36.7 (−50.7, −22.7)	−5.163	0.597 *	−26.2 (−43.7, −8.7)	−2.961	−0.024 *	17.5 (−1.7, 36.7)	1.791	0.300

* *p* < 0.05; ^c^ AQI scores during the outbreak were lower than before the outbreak. Category A—1 month to 3 months before the outbreak (October 2019 to December 2019); Category B—3 months to 6 months before the outbreak (July 2019 to September 2019); Category C—6 months to 9 months before the outbreak (April 2019 to June 2019); Category D—9 months to 12 months before the outbreak (January 2019 to March 2019); Category K (constant)—during the outbreak (January 2020 to March 2020).

## Data Availability

Not applicable.

## Supplementary Materials

The following are available online at https://www.mdpi.com/1660-4601/18/6/3172/s1, Table S1: Descriptive statistics for key air pollutants in ten mega cities during the COVID-19 outbreak, Table S2: Descriptive statistics for key air pollutants in ten mega cities before the COVID-19 outbreak, Table S3: Significant mean difference between air pollutants concentration level during and prior to the COVID-19 outbreak (independent t test result).
