# Supplementary material for: Air Pollution Status in 10 Mega-Cities in China during the Initial Phase of the COVID-19 Outbreak
Source: Int J Environ Res Public Health. 2021 Mar 19;18(6):3172. doi: 10.3390/ijerph18063172 (PMC8003380; doi:10.3390/ijerph18063172)
Supplement: Supplementary file 1 [file ijerph-18-03172-s001.pdf]

International Journal of
Environmental Research and Public Health

MDPI

Table S1: Descriptive statistics for key air pollutants in ten mega cities during the covid-19 outbreak.

	Mean(SD)	Median	Min	Max	IQR	Range	P25	P75	Skewness	Kurtosis
**Fine Particulate matter ($PM_{2.5}$) μg/m³**										
Beijing	51.7(46.8)	41	3	207	48	204	17	65	1.59	2.15
Shanghai	37.9(26.4)	30	8	131	30	123	18	48	1.61	2.51
Xi'an	83.8(52.8)	68	16	228	72	212	44	116	1.01	0.28
Chongqing	45.4(16.4)	42	15	90	20	75	35	55	0.65	0.05
Wuhan	44.1(24.3)	41	8	108	28	100	28	56	0.89	0.34
Guangzhou	24.7(11.3)	25	4	69	17	65	16	33	0.57	1.33
Chengdu	53.6(25.0)	49	10	119	40	109	35	75	0.53	-0.41
Harbin	85.9(67.6)	61	12	277	90	265	35	125	1.19	0.63
Tianjin	68.4(57.3)	47	6	239	66	233	26	92	1.40	1.46
Shenzhen	22.5(8.7)	21	4	48	11	44	17	28	0.46	0.36
**Inhalable Particulate matter (PM_{10}) μg/m³**										
Beijing	58.5(37.3)	51	8	161	47	153	28	75	1.09	0.63
Shanghai	39.7(20.5)	36	7	106	28	99	24	52	1.00	0.79
Xi'an	117.5(53.7)	120	24	294	72	270	71	143	0.58	0.43
Chongqing	63.5(21.4)	62	21	126	30	105	47	77	0.53	0.39
Wuhan	56.7(28.5)	53	12	135	46	123	32	78	0.52	-0.35
Guangzhou	42.4(21.6)	40	6	136	26	130	28	54	0.99	2.79
Chengdu	77.1(31.6)	70	19	134	55	115	49	104	0.19	-1.12
Harbin	96.4(66.3)	73	20	284	96	264	45	141	1.12	0.53
Tianjin	78.1(51.6)	65	9	227	55	218	42	97	1.18	0.90

Shenzhen	37.7(14.9)	36	6	79	19	73	29	48	0.45	0.24
**Sulfur dioxide (SO_2) μg/m³**										
Beijing	4.8(2.8)	4	2	13	4	11	3	7	1.10	0.48
Shanghai	6.3(1.9)	6	4	13	2	9	5	7	1.27	1.46
Xi'an	11.0(3.7)	11	5	21	5	16	8	13	0.72	0.49
Chongqing	7.5(1.8)	7	5	13	3	8	6	9	0.84	0.23
Wuhan	7.7(2.7)	7	5	17	3	12	6	9	1.24	1.45
Guangzhou	6.0(1.2)	6	4	10	2	6	5	7	0.65	0.87
Chengdu	6.8(1.7)	6	5	13	2	8	6	8	1.29	1.70
Harbin	28.6(13.2)	26	9	56	22	47	17	39	0.48	-1.01
Tianjin	9.8(4.6)	9	3	28	6	25	6	12	1.08	2.07
Shenzhen	5.3(0.5)	5	5	7	1	2			1.35	0.60
**Carbon monoxide (CO) mg/m³**										
Beijing	0.8(0.5)	0.7	0	2	1	2	0.40	1.00	1.29	1.50
Shanghai	0.7(0.2)	0.6	0	1	0	1	0.50	0.80	1.21	1.39
Xi'an	1.0(0.5)	0.9	0	2	1	2	0.60	1.40	0.57	-0.63
Chongqing	0.9(0.2)	0.8	0	1	1	0	0.70	1.00	-0.17	-0.44
Wuhan	1.0(0.2)	1.0	1	2	0	1	0.80	1.10	0.25	-0.02
Guangzhou	0.8(0.2)	0.8	1	1	0	1	0.70	0.90	0.69	-0.01
Chengdu	0.8(0.2)	0.8	0	1	0	1	0.60	0.90	0.57	-0.06
Harbin	0.9(0.4)	0.8	0	2	1	2	0.6	1.20	0.96	0.08
Tianjin	1.0(0.6)	0.9	0	3	1	2	0.60	1.30	1.07	0.68
Shenzhen	0.6(0.1)	0.6	1	1	0	1	0.50	0.70	0.95	0.65
**Nitrogen dioxide (NO_2) μg/m³**										

Beijing	30.8(15.4)	29	5	66	19	61	19	38	0.48	-0.37
Shanghai	35.1(13.6)	33	15	76	20	61	24	44	0.75	-0.04
Xi'an	41.2(16.6)	40	4	82	28	78	27	55	0.05	-0.56
Chongqing	33.0(11.4)	33	9	62	17	53	24	41	0.38	-0.31
Wuhan	26.7(12.6)	23	10	76	16	66	17	33	1.37	2.12
Guangzhou	37.6(17.8)	36	9	104	21	95	27	48	0.82	1.25
Chengdu	34.6(16.7)	33	8	85	25	77	20	45	0.53	-0.39
Harbin	37.7(20.7)	30	12	90	27	78	23	50	1.02	-0.06
Tianjin	43.9(21.0)	39	9	109	23	100	31	54	0.96	0.85
Shenzhen	21.9(8.6)	21	10	50	11	40	15	26	0.88	0.66
				**Ozone (O_3) μg/m³**						
Beijing	68.0(22.8)	69	13	131	24	118	56	80	-0.18	0.37
Shanghai	84.1(23.9)	84	27	164	35	137	66	101	0.20	0.71
Xi'an	73.0(32.8)	74	7	144	48	137	47	95	-.015	-0.51
Chongqing	51.8(31.8)	45	9	159	35	150	24	41	1.33	1.63
Wuhan	71.1(30.3)	72	5	136	45	131	51	96	-0.08	-0.32
Guangzhou	75.7(34.5)	72	7	184	55	177	48	103	0.43	-0.22
Chengdu	70.2(31.1)	71	14	157	36	143	50	86	0.51	0.69
Harbin	80.9(23.4)	81	33	156	29	123	66	95	0.13	0.38
Tianjin	68.9(24.4)	75	10	118	28	108	56	84	-0.50	0.02
Shenzhen	76.6(20.2)	76	22	129	30	107	62	92	-0.09	-0.33

Table S2: Descriptive statistics for key air pollutants in ten mega cities before the covid-19 outbreak.

		1 - 3 months b/o				3 - 6 months b/o				6 - 9 months b/o				9 - 12 months b/o			
		**Mean**	**Med**	**Min**	**Max**	**Mean**	**Med**	**Min**	**Max**	**Mean**	**Med**	**Min**	**Max**	**Mean**	**Med**	**Min**	**Max**
**$PM_{2.5}$ μg/m³**	Beijing	42.5	37	6	183	31.8	27	3	83	41.3	38	5	106	60.0	39	4	217
	Shanghai	36.1	32	10	113	23.7	21.5	6	54	33.8	30	12	93	47.2	43	10	122
	Xi'an	72.5	57	9	269	25.2	24	6	57	37.5	32	7	170	98.5	76	20	296
	Chongqing	41.6	38.5	10	114	25.0	22	10	62	28.9	29	12	56	56.5	53.5	19	132
	Wuhan	49.0	46	7	136	29.3	27.5	15	51	34.5	34	7	69	69.0	61	16	164
	Guangzhou	41.2	40	15	76	23.8	22.5	6	57	20.0	19	8	44	34.4	29	9	92
	Chengdu	52.0	44.5	7	138	26.5	24	4	77	32.1	28	5	76	59.6	57	17	116
	Harbin	48.7	33.5	5	248	15.6	13	4	35	24.3	20	4	81	77.6	58.5	14	371
	Tianjin	54.3	42.5	10	175	36.0	32	6	86	44.0	39	8	124	70.6	56	10	248
	Shenzhen	35.7	36	17	69	18.5	15	5	60	15.6	15	6	49	26.9	25	9	74
**PM_{10} μg/m³**	Beijing	67.7	57	15	188	48.1	46.5	11	99	76.4	68	15	292	77.5	66	19	227
	Shanghai	54.9	44	14	212	32.5	32	10	66	45.3	39	15	111	59.1	44	18	127
	Xi'an	114.0	102.5	19	296	53.0	50.5	11	112	92.4	71	14	581	150.3	138	58	339
	Chongqing	62.7	57	17	156	42.5	57	21	99	48.5	48	16	94	80.0	76.5	26	177
	Wuhan	80.5	77	15	179	54.3	53	23	97	66.0	62	13	173	89.8	85.5	21	164
	Guangzhou	77.9	75.5	36	154	43.1	39	3	95	36.4	35	12	82	54.7	49	14	125
	Chengdu	77.7	69.5	13	178	46.2	42	9	128	56.3	52	9	128	91.3	88.5	26	165
	Harbin	69.9	52	19	273	36.1	33	9	84	69.0	45	10	559	105.5	92.5	33	310
	Tianjin	83.1	72	24	240	56.2	56	10	109	82.4	70	22	407	100.3	87.5	21	287
	Shenzhen	64.3	63	28	114	32.8	28	13	87	26.9	26	14	61	42.9	40	14	119
**S**	Beijing	3.9	3	2	15	2.7	3	2	7	4.3	3	2	12	6.9	6	2	21

	Shanghai	8.1	8	5	15	5.6	5.5	4	10	6.3	6	4	11	7.5	7	5	16
	Xi'an	10.6	9.5	5	22	5.4	5	4	11	5.8	5	3	13	12.3	10.3	4	32
	Chongqing	7.5	7	4	14	7.1	7	4	12	6.6	6	4	13	8.6	8	5	14
	Wuhan	10.4	10	6	24	8.5	8	4	18	7.8	7	4	15	8.8	8	5	17
	Guangzhou	9.3	9	6	15	6.6	6	4	13	5.8	6	4	9	6.0	6	4	10
	Chengdu	7.1	7	4	13	5.5	5	4	14	6.3	6	4	14	6.8	7	3	12
	Harbin	19.3	17	7	49	8.3	8	6	14	10.8	10	6	20	27.8	27	12	48
	Tianjin	10.9	11	5	24	7.6	8	3	20	10.2	10	4	19	14.7	14	4	36
	Shenzhen	6.8	6.	4	12	4.6	4	3	9	4.1	4	3	5	5.1	5	3	8
CO) mg/m^3	Beijing	08	0.8	0	2	0.6	0.6	0	1	0.6	0.6	0	1	0.8	0.8	0	3
	Shanghai	0.7	0.6	0	1	0.6	0.6	0	1	0.6	0.6	0	1	9.8	0.7	0	1
	Xi'an	1.0	0.9	0	2	0.6	0.6	0	1	0.6	0.7	0	1	1.2	1.2	0	3
	Chongqing	0.9	0.8	1	1	0.6	0.6	0	1	0.6	0.6	0	1	1.0	1.0	1	2
	Wuhan	1.0	1.0	1	2	0.9	0.9	1	1	1.0	0.9	1	2	1.1	1.1	1	2
	Guangzhou	0.9	0.9	1	1	0.7	0.7	1	1	0.7	0.7	1	1	1.0	1.0	1	2
	Chengdu	0.8	0.7	1	1	0.8	0.8	1	1	0.8	0.8	1	1	1.0	1.0	1	2
	Harbin	0.8	0.7	0	2	0.6	0.6	0	1	0.6	0.6	0	1	1.1	1.0	1	2
	Tianjin	1.0	0.9	0	3	0.9	0.9	0	2	0.8	0.8	0	2	1.2	1.1	0	3
	Shenzhen	0.7	0.6	0	1	0.6	0.5	0	1	0.6	0.6	0	1	0.8	0.7	1	1
NO_2 μg/m^3	Beijing	44.6	44	4	94	28.4	28	15	45	31.6	30	14	54	41.4	40	9	101
	Shanghai	50.4	46.5	16	115	30.0	28.5	6	63	37.3	36	17	77	48.5	48	14	109
	Xi'an	56.3	56.5	20	92	35.6	35	4	70	41.1	40	18	76	57.7	58.5	24	98
	Chongqing	41.1	41	21	72	33.4	31.5	18	73	36.2	35	20	64	41.8	42	16	75
	Wuhan	54.2	47.5	18	109	33.2	30	15	86	41.0	37	15	80	48.6	45	13	88

	Guangzhou	55.6	49.5	23	123	36.2	35	2	67	40.0	39	18	68	47.4	45	15	107
	Chengdu	44.6	44	25	71	38.0	36.5	13	63	37.0	37	12	72	48.3	48.5	14	84
	Harbin	36.8	32	12	82	25.7	26	12	50	26.5	26	12	55	41.1	39	15	75
	Tianjin	53.7	53.5	18	96	30.8	31	12	63	33.4	32	17	60	50.8	50	9	102
	Shenzhen	32.0	30.5	12	72	21.7	20	7	44	21.0	20	8	39	26.8	24.5	10	73
O_3 μg/m³	Beijing	37.1	37	0	146	142.9	148	38	243	141.6	126	39	260	65.2	66.5	12	131
	Shanghai	80.5	74.5	16	171	111.6	115.5	24	246	120.7	115	43	274	78.9	79.5	16	156
	Xi'an	37.4	33.5	7	139	133.3	133	56	214	120.9	123	22	218	62.4	64.5	13	112
	Chongqing	32.3	26	5	153	125.4	124	23	277	100.8	88	31	218	49.2	41	9	160
	Wuhan	71.3	61.5	10	216	148.2	149	69	236	126.1	129	28	105	54.5	49	11	124
	Guangzhou	126.8	121	14	229	126.8	126	25	232	88.7	85	18	233	62.3	50.5	6	161
	Chengdu	43.7	41	7	105	110.0	99.5	33	250	119.2	114	47	236	59.2	59	13	152
	Harbin	49.4	45	25	185	77.2	76.5	13	163	100.9	95	25	199	65.1	65	33	124
	Tianjin	51.3	48	6	192	154.0	157	32	256	150.8	137	52	286	68.5	69	12	140
	Shenzhen	121.2	121.5	46	245	103.2	79.5	33	241	78.2	71	37	171	72.4	70	16	129

Table S3: Significant mean difference between air pollutants concentration level during and prior to the covid-19 outbreak (independent t test result).

City	Air pollutant	Comparison	Mean diff (95% CI)	t value	Effect size	City	Air pollutant	Comparison	Mean diff (95% CI)	t value	Effect size
Beijing	$PM_{2.5}$	B	-19.9(-30.4, -9.5)	-3.757	-0.555	Guangzhou	$PM_{2.5}$	A	16.5(13.0, 20.0)	9.398	1.389
	PM_{10}	B	-10.4(-19.3, -1.5)	-2.304	-0.340			C	-4.7(-7.5, -1.9)	-3.293	-0.488
		C	17.9(5.9, 30.0)	2.909	0.431			D	9.7(5.2, 14.1)	4.305	0.640
		D	19.0(6.7, 31.4)[c]	3.042	0.452		PM_{10}	A	35.5(28.5, 42.3)	10.101	1.493

	SO_2	A	-0.9(-1.7, -0.2)	-2.438	-0.359
		B	-2.1(-2.7, -1.5)	-6.867	-1.013
		D	2.1(1.1, 3.1)[c]	4.258	0.634
	CO	B	-0.12(-0.3, -0.02)	-2.428	-0.365
		C	-0,2(-0.3, -0.1)	-3.176	-0.459
	NO_2	A	13.9(8.9, 18.3)[c]	5.487	0.811
		D	10.6(5.2, 16.0)[c]	3.885	0.578
	O_3	A	-30.9(-37.7, -24.1)	-8.990	-1.329
		B	74.9(62.9, 86.9)[c]	12.346	1.825
		C	73.6(61.2, 85.9)[c]	11.728	1.739
Shanghai	$PM_{2.5}$	B	-14.2(-20.2, -8.2)	-4.671	-0.691
		D	9.3(1.5, 17.1)[c]	2.338	0.348
	PM_{10}	A	15.2(6.2, 24.2)[c]	3.327	0.492
		B	-7.2(-12.1, -2.3)	-2.883	-0.426
		D	19.5(12.3, 26.7)[c]	5.327	0.792
	SO_2	A	1.8(1.2, 2.4)[c]	5.818	0.856
		B	-0.7(-1.1, -0.2)	-2.943	-0.438
		D	1.3(0.6, 1.9)[c]	3.783	0.559
	CO	B	-0.12(-0.17, -0.05)	-3.669	-0.550
		C	-0.06(-0.13, -0.01)	-2.285	-0.348
		D	0.09(0.02, 0.16)[c]	2.505	0.378
	NO_2	A	15.3(10.3, 20.2)[c]	6.070	0.897
		B	-5.4(-8.9, -1.9)	-3.055	-0.452
		D	13.4(8.6, 18.3)[c]	5.450	0.810
	O_3	B	27.5(16.7, 38.4)[c]	5.004	0.740
		C	36.7(26.3, 47.0)[c]	6.977	1.034
		C	-6.0(-11.3, -0.8)	-2.278	-0.388
		D	12.3(5.0, 19.6)	3.319	0.493
	SO_2	A	3.4(2.9, 3,8)	14.306	2.115
		C	-6.0(-11.3, -0.8)	-2.278	-0.188
	CO	A	0.1(0.03, 0.13)	3.156	0.425
		B	-0.1(-.13, -0.05)	-4.176	-0.647
		C	-0.05(-0.09, -0.01)	-2.595	-0.420
		D	0.2(0.15, 0.27)	6.744	0.996
	NO_2	A	18.0(12.0, 24.0)	5.860	0.866
		D	9.8(4.5, 15.1)	3.667	0.545
	O_3	A	51.1(38.6, 63.7)	8.030	1.187
		B	51.1(37.7, 64.5)	7.522	1.112
		C	13.0(1.7, 24.3)	2.276	0.337
		D	-13.4(-24.6, -2.2)	-2.365	-0.352
CHENGDU	$PM_{2.5}$	B	-27.1(-33.0, -21.2)	-9.020	-1.333
		C	-21.5(-27.5, -15.4)	-6.997	-1.037
	PM_{10}	B	-30.8(-39.2, -22.7)	-7.446	-1.101
		C	-20.8(-29.3, -12.3)	-4.822	-1.037
		D	14.3(5.0, 23.5)	3.030	0.450
	SO_2	B	-1.3(-1.8, -0.8)	-5.224	-0.776
	CO	D	0.17(0.11, 0.23)	5.272	0.780
	NO_2	D	13.7(9.2, 18.2))[c]	6.043	0.899
	O_3	B	39.8(27.6, 51.9)	6.448	0.953
		C	49.0(38.1, 59.9)	8.876	1.316
		D	-11.0(-19.7, -2.3)	-2.500	-0.372
H A	$PM_{2.5}$	A	-37.2(-54.3, -20.1)	-4.291	-0.634

**Xi'an**	$PM_{2.5}$	B	-58.7(-69.7, -49.6)	-10.462	-1.574			B	-70.3(-84.3, -56.3)	-9.914	-1.466
		C	-46.4(-58.5, -34.2)	-7.539	-1.118			C	-61.61(-75.9, -47.3)	-8.479	-1.257
	PM_{10}	B	-64.5(-76.3, -52.7)	-10.786	-1.595		PM_{10}	A	-26.5(43.6, -9.4)	-3.059	-0.452
		C	-25.1(-45.5, -4.8)	-2.436	-0.361			B	-60.3(-74.3, -46.3)	-8.476	-1.253
		D	32.8(16.1, 49.6)[c]	3.866	0.575			C	-27.4(-48.3, -6.6)	-2.597	0.385
	SO_2	B	-5.5(-6.3, -4.7)	-13.449	-1.991		SO_2	A	-9.3(-12.8, -5.9)	-5.324	-0.787
		C	5.2(-6.1, -4.3)	-11.948	-1.772			B	-20.3(-23.0, -17.6)	-14.618	-2.161
	CO	B	-0.4(-0.5, -0.3)	-7.327	-1.081			C	-17.9(-20.7, -15.0)	-12.561	-1.863
		C	-0.4(-0.5, -0.3)	-6.766	-0.989		CO	A	-0.1(-0.3, -0.03)	-2.684	-0.387
		D	0.2(0.06, 0.3)[c]	2.835	0.425			B	-0.3(-0.4, -0.2)	-7.440	-1.093
	NO_2	A	15.1(10.3, 19.8)[c]	6.150	0.909			C	-0.4(-0.4, -0.3)	-8.132	-1.211
		D	16.6(11.6, 21.6)[c]	6.561	0.975			D	0.1(0.03, 0.25)[c]	2.487	0.369
	O_3	A	-35.6(-43.6, -27.6)	-8.789	-1.300		NO_2	B	-12.0(-16.5, -7.5)	-5.277	-0.780
		B	60.3(49.2, 71.4)[c]	10.721	1.585			C	-11.2(-15.8, -6.6)	-4.833	-0.716
		C	47.9(36.3, 59.5)[c]	8.168	1.211		O_3	A	-31.5(-38.2, -24.8)	-9.270	-1.371
		D	-10.6(-19.5, -1.7)	-2.352	-0.350			C	20.0(11.7, 28.3)[c]	4.761	0.706
								D	-15.8(-22.1, -9.5)	-4.939	-0.724
**Chongqing**	$PM_{2.5}$	B	-20.4(-24.4, -16.3)	-9.896	-1.467						
		C	-16.5(-20.3, -12.7)	-8.499	-1.260	**Tianjin**	$PM_{2.5}$	B	-32.4(-44.7, -20.1)	-5.195	-0.768
		D	11.1(5.0, 17.3)[c]	3.597	0.535			C	-24.4(-37.2, -11.6)	-3.761	-0.558
	PM_{10}	B	-21.1(-26.7, -15.34)	-7.331	-1.084		PM_{10}	B	-22.0(-33.4, -10.5)	-3.771	-0.558
		C	-16.5(-20.4, -12.7)	-8.499	-0.780			D	22.3(5.8, 38.7)[c]	2.664	0.396
		D	11.1(5.0, 17.3)[c]	3.597	0.591		SO_2	B	-2.1(-3.3, -1.0)	-3.581	-0.530
	SO_2	B	-21.1(-26.7, -15.4)	-7.331	-0.455			D	5.0(3.2, 6.7)[c]	5.515	0.819
		C	-0.9(-1.4, -0.3)	-3.095	-0.535		CO	B	-0.2(-0.3, -0.03)	-2.519	-0.380
		D	1.2(0.6, 1.8)[c]	3.762	0.559			C	-0.3(-0.4, -0.1)	-4.085	-0.620
	CO	B	-0.09(-0.14, -.05)	-4.031	-0.576		NO_2	A	9.8(4.1, 15.6)[c]	3.399	0.503
		C	-.05(-0.1, -.0.01)	-2.311	-0.317			B	-13.1(-17.9, -8.2)	-5.333	-0.778

**Wuhan**		D	0.1(0.04, 0.2)[c]	3.282	0.512
	NO_2	A	8.4(5.1, 11.7)[c]	5.078	0.517
		C	3.2 (0.2, 6.2)[c]	2.072	0.307
		D	8.8(5.5, 12.2)[c]	5.179	0.770
	$PM_{2.5}$	C	-9.7(-15.4, -4.0)	-3.355	-0.497
		D	24.8(16.4, 33.3)[c]	5.799	0.862
	PM_{10}	A	23.8(14.5, 33.1)[c]	5.054	0.747
		C	9.3(0.8, 17.8)[c]	2.166	0.321
		D	33.1(23.7, 42.5)[c]	6.956	1.034
	SO_2	A	2.7(1.8, 3.6)[c]	5.747	0.850
		D	1.1(0.2, 2.0)[c]	2.457	0.366
	CO	D	0.2(0.09, 0.3)[c]	4.162	0.637 l
	NO_2	A	27.5(22.1, 40.0)[c]	9.985	1.476
		B	6.6(2.7, 10.4)[c]	3.351	0.496
		C	14.3(10.3, 18.3)[c]	7.082	1.050
		D	21.9(17.4, 26.4)[c]	9.534	1.417
	O_3	B	77.1(66.4, 87.7)[c]	14.271	2.110
		C	55.2(44.2, 70.0)[c]	9.961	1.476
		D	-16.5(-25.3, -7.7)	-3.705	-0.551
**SHENZHEN**		C	-10.6(-15.4, -5.7)	-4.307	-0.639
		D	6.9(0.7, 13.2)[c]	2.205	0.328
	O_3	A	-17.7(-26.3, -9.1)	-4.056	-0.600
		B	85.1(72.8, 97.3)[c]	13.709	2.027
		C	81.9(69.5, 94.3)[c]	13.044	1.934
	$PM_{2.5}$	B	-58.7(-69.7, -47.6)	-10.462	-0.405
		C	-46.4(-58.5, -34.2)	-7.539	-0.877
	PM_{10}	B	-64.5(76.3, -52.7)	-10.786	-0.307
		C	-25.1(-45.5, -4.8)	-2.436	-0.858
		D	32.8(16.1, 49.6)[c]	3.866	0.293
	SO_2	B	-5.5(-6.3, -4.7)	-13.449	-0.703
		C	-5.2(-6.0, -4.3)	-11.948	-2.855
	CO	B	-0.4(-0.5, -0.3)	-7.327	-0.580
		C	-0.4(-0.5, -0.3	-6.766	-0.580
		D	0.2(0.1, 0.3)[c]	2.835	0.833
	NO_2	A	15.1(10.3, 20.0)	6.150	0.958
		B	-5.5(-9.8, -1.3)	-2.571	-0.209
		D	16.6(11.6, 21.6)[c]	6.561	0.479
	O_3	A	-35.6(43.6, -27.6)	-8.789	1.451
		B	60.3(5.6, 49.2)[c]	10.721	0.622
		C	47.9(36.4, 59.5)[c]	8.168	0.060
		D	-10.6(-19.5, -1.7)	-2.352	-0.175

A - 1 to 3 months before the outbreak vs during the outbreak; B - 3 to 6 months before the outbreak vs during the outbreak;
C - 6 to 9 months before the outbreak vs during the outbreak; D - 9 to 12 months before the outbreak vs during the outbreak;
[c] means during the outbreak was lower than before the outbreak.
